# LKB1 Is Required for the Development and Maintenance of Stereocilia in Inner Ear Hair Cells in Mice

**DOI:** 10.1371/journal.pone.0135841

**Published:** 2015-08-14

**Authors:** Yuqin Men, Aizhen Zhang, Haixiang Li, Tingting Zhang, Yecheng Jin, Huashun Li, Jian Zhang, Jiangang Gao

**Affiliations:** 1 Institute of Developmental Biology, School of Life Science, Shandong University, Jinan, Shandong, China; 2 SARITEX Center for Stem Cell, Engineering Translational Medicine, Shanghai East Hospital, Advanced Institute of Translational Medicine, Tongji University School of Medicine, Shanghai, China; 3 Center for Stem Cell&Nano-Medicine, Shanghai Advanced Research Institute, Chinese Academy of Sciences, Shanghai, China; 4 Shenzhen Key Laboratory for Molecular Biology of Neural Development, Shenzhen Institutes of Advanced Technology, Chinese Academy of Sciences, Shenzhen, Guangdong, China; University of Minnesota, UNITED STATES

## Abstract

The LKB1 gene, which encodes a serine/threonine kinase, was discovered to play crucial roles in cell differentiation, proliferation, and the establishment of cell polarity. In our study, LKB1 conditional knockout mice (Atoh1-LKB1^-/-^ mice) were generated to investigate LKB1 function in the inner ear. Tests of auditory brainstem response and distortion product otoacoustic emissions revealed significant decreases in the hearing sensitivities of the Atoh1-LKB1^-/-^ mice. In Atoh1-LKB1^-/-^ mice, malformations of hair cell stereocilliary bundles were present as early as postnatal day 1 (P1), a time long before the maturation of the hair cell bundles. In addition, we also observed outer hair cell (OHC) loss starting at P14. The impaired stereocilliary bundles occurred long before the presence of hair cell loss. Stereociliary cytoskeletal structure depends on the core actin-based cytoskeleton and several actin-binding proteins. By Western blot, we examined actin-binding proteins, specifically ERM (ezrin/radixin/moesin) proteins involved in the regulation of the actin cytoskeleton of hair cell stereocilia. Our results revealed that the phosphorylation of ERM proteins (pERM) was significantly decreased in mutant mice. Thus, we propose that the decreased pERM may be a key factor for the impaired stereocillia function, and the damaged stereocillia may induce hair cell loss and hearing impairments. Taken together, our data indicates that LKB1 is required for the development and maintenance of stereocilia in the inner ear.

## Introduction

Sound transduction initiates in the external auditory canal and leads to the vibration of the tympanic membrane or the eardrum. Compression of the tympanic membrane transmits sound energy to the cochlea of the inner ear, a fluid-filled, spiral shaped structure for auditory detection. Within the cochlea lies the Organ of Corti (OC), which serves as one of the core components for auditory signal transduction. The OC comprises of mechanoreceptors in the form of hair cells (HCs) with a single row of inner hair cells (IHCs) and three rows of outer hair cells (OHCs) [1]. Hair cells contain hairlike stereocilia that transmits sound signals based on the movement of the tectorial membrane, leading to the release of the neurotransmitter glutamate. This cascade results in activation of afferent neurons collectively known as the cochlear branch of the vestibulocochlear nerve that feeds into the auditory cortex.

Over the years, HCs have been a topic of interests as its loss results in the lack of hearing observed in presbycusis, head trauma, and a side effect of chemotherapy. An important structure on the apical surface of each hair cell is hair bundles divided into two types: actin-based stereociliary bundle and a single tubulin-based kinocilum [2, 3]. Another critical part is a specialized actin network known as the cuticular plate, which is located on the apical membrane. The cuticular plate consists of sterocilia actin filaments formed rootlets that hold as an anchor for the stereocilia [4, 5]. In the hearing process, the development and maintenance of these actin structures is crucial to sustain the viability and function of inner ear hair cells The abnormality of these actin-based cytoskeleton structures in the hair cell, particularly those of the stereocilia [6–8] and the rootlets [9], is often the root cause of hearing loss.

The liver kinase B1 (LKB1) gene is known as an important serine/threonine kinase11 (STK11) and potent tumor suppressor. LKB1, which encodes a 48-kDa protein, was identified and characterized as a novel gene encoding for the serine/threonine kinase within a region on chromosome 19p13.3. This region was identified as a locus for Peutz-Jeghers syndrome (PJS). LKB1 contains a nuclear localization signal domain, which is potentially suggests that LKB1 is normally localized in the nucleus [10]. The scaffold protein Mo25 binds to the pseudokinase STE20-related adaptor (STRAD) and LKB1 to activate a LKB1/STRAD/Mo25 ternary complex. The activation of LKB1 is associated with its translocation to the cytoplasm [11, 12]. LKB1 has been implicated in the control of a variety of functions, ranging from proliferation and migration to senescence, apoptosis, DNA damage response and differentiation *in vitro*.

LKB1 is also considered to be a critical tumor suppressor gene frequently mutated in tumors in various tissues such as lung and cervical cancer [13, 14]. Heterozygous inactivating germline mutations in LKB1 were detected to cause a predisposition for PJS. Additionally, in a variety of sporadic malignancies, LKB1 is associated with tumor progression as seen in colorectal, pancreatic, and breast cancer [15, 16]. These studies reveal a strong association between LKB1 mutations and increased risk with oncogenic events.

In mammals, LKB1 is expressed ubiquitously in various tissues, particularly the pancreas, liver, testes, skeletal muscles, and nervous system. In view of the wide expression of the LKB1 gene, conventional LKB1 gene knockout results in embryonic lethality on embryonic day(E) 8–9, highlighting its crucial role during early embryonic development [17, 18]. Analysis of the embryos revealed severe developmental defects, including impaired neural tube closure and somitogenesis, mesenchymal tissue cell death, defective vasculature, and deformation of extra-embryonic tissue. To examine further the tissue specificity of LKB1 functions *in vivo* during embryonic development and adult maturation, various tissue-specific conditional knockout mouse models were constructed [18–22]. Using these knockout mouse models, it was reported that LKB1 plays crucial roles in multiple tissues of mammals, affecting cell polarity, energy metabolism, embryonic growth, development, and cell differentiation.

In previous studies, the wide expression and critical function of LKB1 were demonstrated. Based on these findings from these previous studies and our preliminary findings in the expression of LKB1, we decided to examine the function of LKB1 in the inner ear. In our study, LKB1 conditional knockout mice in the inner ear were generated by crossing LKB1^LoxP/LoxP^ mice with Atoh1-Cre mice. In this study, our results showed that the LKB1-deficient mice displayed impaired hearing and malformations of the stereociliary bundles of cochlear hair cells. Our findings suggest that LKB1 plays an essential role in maintaining normal hearing by regulating the development and maintenance of stereocilia in inner ear hair cells.

## Materials and Methods

### Ethics Statement

All animal experimental procedures were approved by Ethics Committee of Shandong University. Animal management was performed strictly in accordance with the standards of the Animal Ethics Committee of Shandong University (Permit Number: ECAESDUSM 20123004).

### Animals

The LKB1 conditional allele, Atoh1-Cre mice were used as a result of FVB genetic background. Mice homozygous for floxed LKB1 exon3-6 (LKB1^LoxP/LoxP^) (MGI accession number: 4440829) [23] were crossed with Atoh1-Cre mice (MGI accession number: 4455013) [24]. Atoh1-Cre activities were confined to the developing inner ear, the hindbrain, the spinal cord, and the intestine. In the inner ear, Atoh1-Cre activities were detected in nearly all of the hair cells and in certain supporting cells.

We genotyped wild-type mice (WT), heterozygous mice and homozygous mice by PCR. DNA was extracted from mice tail snips for PCR analysis. Mice were genotyped for Cre using the following primers: Cre-F (5′-AGCTAAACATGCTTCATCGTCGGTC-3′) and Cre-R (5′-TATCCAGGTTACGGATATAGTTCATG-3′). The PCR genotyping of the floxed LKB1 allele was detected by the following primers: LKB1-L (5’-ATCGGAATGTGATCCAGCTT-3’) and LKB1-R (5’-GAGATGGGTACCAGGAGTTGGGGCT-3’).

### Auditory brainstem responses (ABR) and distortion product oto acoustic emissions (DPOAEs)

ABR was performed as described previously [25]. ABR was measured in a soundproof room. Mice were anesthetized at P30 with 0.007g/ml pentobarbital sodium. Three electrodes were inserted in the mice through the cranial vertex, the external ear, and the rear region above the tail respectively. ABR tone pips of 4 kHz, 8 kHz, 12 kHz, 16 kHz, 24 kHz and 32 kHz were generated using a Tucker Davis Technologies (TDT) workstation running SigGen32 software (TDT). Auditory thresholds were determined by decreasing the sound intensities from 90 to 10 dB until lowest sound intensity at which reproducible waves could be recognized was reached. DPOAE response thresholds were tested as described previously [25]. The DPOAE responses at diction frequency 2f1-f2 were measured with two primary tone frequencies (f1 and f2, with f2/f1 = 1.2 and f2 level 10dB < f1level) to predict auditory thresholds. DPOAE response thresholds were recorded at a range of frequencies (4 kHz, 8 kHz, 12 kHz, 16 kHz, 24 kHz and 32 kHz) within the acoustic microphone probe and the TDT system. Emissions *f*1 and *f*2 stimulated the cochlea and passed through a multifunction processor (TDT) to a computer controlled programmable attenuator, buffer amplifier, and earphone. Stimuli were generated digitally and the maximum level of stimuli for DPOAE was 80 dB SPL. 2f1-f2 DPOAE amplitude and the surrounding noise floor were extracted. Hearing thresholds were defined as the averaged signal for each identified frequency tested and the comparison with the corresponding frequency in controls. Statistical comparisons of means were performed using two-way ANOVA followed by Student’s t test with a Bonferroni correction with n>4 animals per genotype. Mean values were quoted ± SEM where p<0.05 indicates statistical significance.

### Immunostaining analysis

Wild-type and Atoh1-LKB1^-/-^ mice were transcardial perfused with 4% paraformaldehyde (PFA) under pentobarbital sodium anesthesia. Subsequently, cochleae were dissected and fixed in 4% paraformaldehyde for 2 hours at RT. Cochleae from adult mice were also decalcified in 10% EDTA overnight at 4°C. For sectioning, the cochleae samples were equilibrated for 4 hours in 15% sucrose at RT and later in 30% sucrose overnight at 4°C. Finally, the samples were embedded in OCT compound and frozen at -20°C in liquid nitrogen. The tissue blocks were sectioned to 7-μm thickness. For whole mount immunostaining, cochleae were exposed to the sensory epithelium and were dissected into basal, middle, and apical sections. Sections or whole mounts were washed with 10 mM PBS and blocked (10% goat serum) for 30 min at RT. Primary antibodies were diluted in 1% bovine serum albumin, 5% heat-inactivated goat serum, and 0.1% Triton X-100. The samples were incubated with primary antibodies overnight at 4°C. After three washes with 10mM PBS, samples were incubated at RT for 1 hour in secondary antibodies (goat anti-rabbit or rabbit anti-goat Alexa-488 or Alexa-568, 1:500; Invitrogen) diluted in the solution (0.1% bovine serum albumin and 0.1% Triton X-100). Lastly, samples were again washed with 10mM PBS, and rhodamine phalloidin (2ug/ml, Sigma) and nuclear stain (DAPI or PI) were applied to the samples for 15 min at RT followed by a final 10mM PBS wash. Cochleae were imaged with a LSM 700 confocal microscope. The quantitative assessment of the OHC loss was performed as described previously [26]. Primary antibodies used were anti-LKB1 polyclonal antibody (rabbit, 1:200, Upstate), anti-ERM polyclonal antibody (rabbit, 1:200, Epitomics), anti-myosin VIIa polyclonal antibody (rabbit, 1:200, Proteus-Bioscience), anti-prestin (N-20) polyclonal antibody (goat, 1:400, Santa Cruz) and anti-Mst4 polyclonal antibody (rabbit, 1:200, Epitomics).

### Western blot

Wild-type and Atoh1-LKB1^-/-^ mice were sacrificed by cervical dislocation and their cochleae were dissected from the heads of mice. Cochleae proteins were incubated in cell lysis buffer (10 mM Tris, pH = 7.4, 1% Triton X-100, 150 mM NaCl, 1 mM EDTA, 0.2 mM PMSF) and extracted using a homogenizer. The proteins from the samples (40 μg) were subjected to SDS-polyacrylamide gel electrophoresis and blotted onto a polyvinylidene difluoride membrane (PVDF). Western blots were performed as described previously [27]. Primary antibodies used were anti-LKB1 polyclonal antibody (rabbit, 1:500, Bioworlde), anti-ERM polyclonal antibody (rabbit, 1:2000, Epitomics), anti-p-ERM polyclonal antibody (rabbit, 1:500, Epitomics), anti-GAPDH monoclonal antibody (mouse, 1:5000, Millipore), and anti-Mst4 polyclonal antibody (rabbit, 1:1000, Epitomics). All data are presented as means ± SEM and data analyses were performed using the GraphPad Prism 5.0 software. Student’s t-test was used for single factor experiments involving two groups. A significance level was set to p<0.05 for all statistical analyses.

### Scanning electron microscopy

For the SEM images, inner ears from Atoh-LKB1^-/-^ and wild-type mice were dissected after transcardial perfusion with 4% PFA under pentobarbital sodium anesthesia and then immersed in 2.5% glutaraldehyde in 0.1 M phosphate buffer overnight at 4°C. Cochleae were dissected out of the temporal bone, post-fixed in 1% osmium tetroxide, 0.1 M phosphate buffer, dehydrated through a graded ethanol series and then critically point dried. Samples were mounted and sputter coated with gold. After the whole process, stereociliary bundles were examined in the middle basal turns of the cochlea using a Hitachi S-4800 Field-Emission scanning electron microscope.

### Histology analysis

To generate paraffin sections, wild-type and Atoh1-LKB1^-/-^ mice were transcardial perfused with 4% PFA under pentobarbital sodium anesthesia conditions. Subsequently, cochleae were rapidly removed from mice and post-fixed in 4% paraformaldehyde at 4°C overnight. The cochleae were dehydrated via an ethanol series from 30% to 100%. Afterwards, the tissue samples underwent dehydration, clearing, and wax immersion. Finally, the tissues were embedded at the proper orientation in paraffin, sectioned at 7-μm thicknesses and stained using hematoxylin and eosin (HE).

## Results

### Conditional inactivation of LKB1 in the developing inner ear

**Fig 1:**
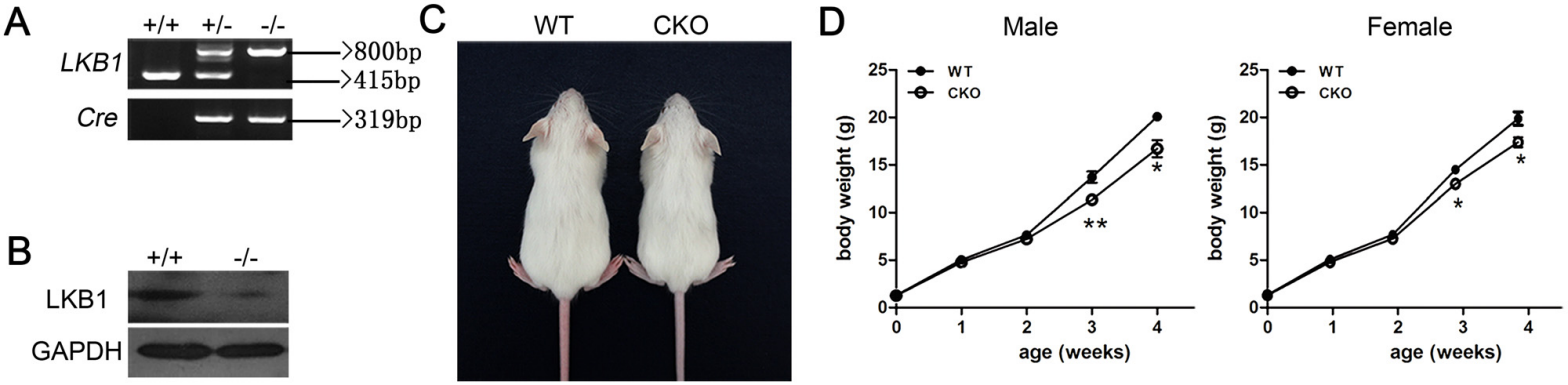
Generation of Atoh1-LKB1^-/-^ conditional knockout mice. (A) Mouse genotyping by PCR analysis. Lanes: wild-type (+/+), heterozygous (+/-) and homozygous (-/-) mice. The LKB1 gene product of the wild-type allele was 415-bp. A 415-bp band and an 800-bp band were detected in the heterozygous mice. Only the 800-bp band was observed in the homozygous mice. The PCR product of the Atoh1-Cre recombinase gene was 319-bp. (B) Western blot analysis of Lkb1 in the cochlea of Atoh1-LKB1^-/-^ and wild-type mice at P14. A 48.6-kD LKB1 protein was decreased obviously in the cochlea of Atoh1-LKB1^-/-^ mice compared with the controls. (C) Gross morphology of wild-type and Atoh1-LKB1^-/-^ mice at P21. There was no obvious difference, except for the smaller size of the mutant mice compared with the controls. (D) The body weight of male and female mice from WT and mutant mice was measured. The mutant mice exhibited significant decreases in weight compared to those of the control group after P21. Error bars indicate SEM. *p<0.05; **p<0.01; ***p<0.001 by Student’s t-test. n = 5 mice per experiment and genotype.

**Fig 2:**
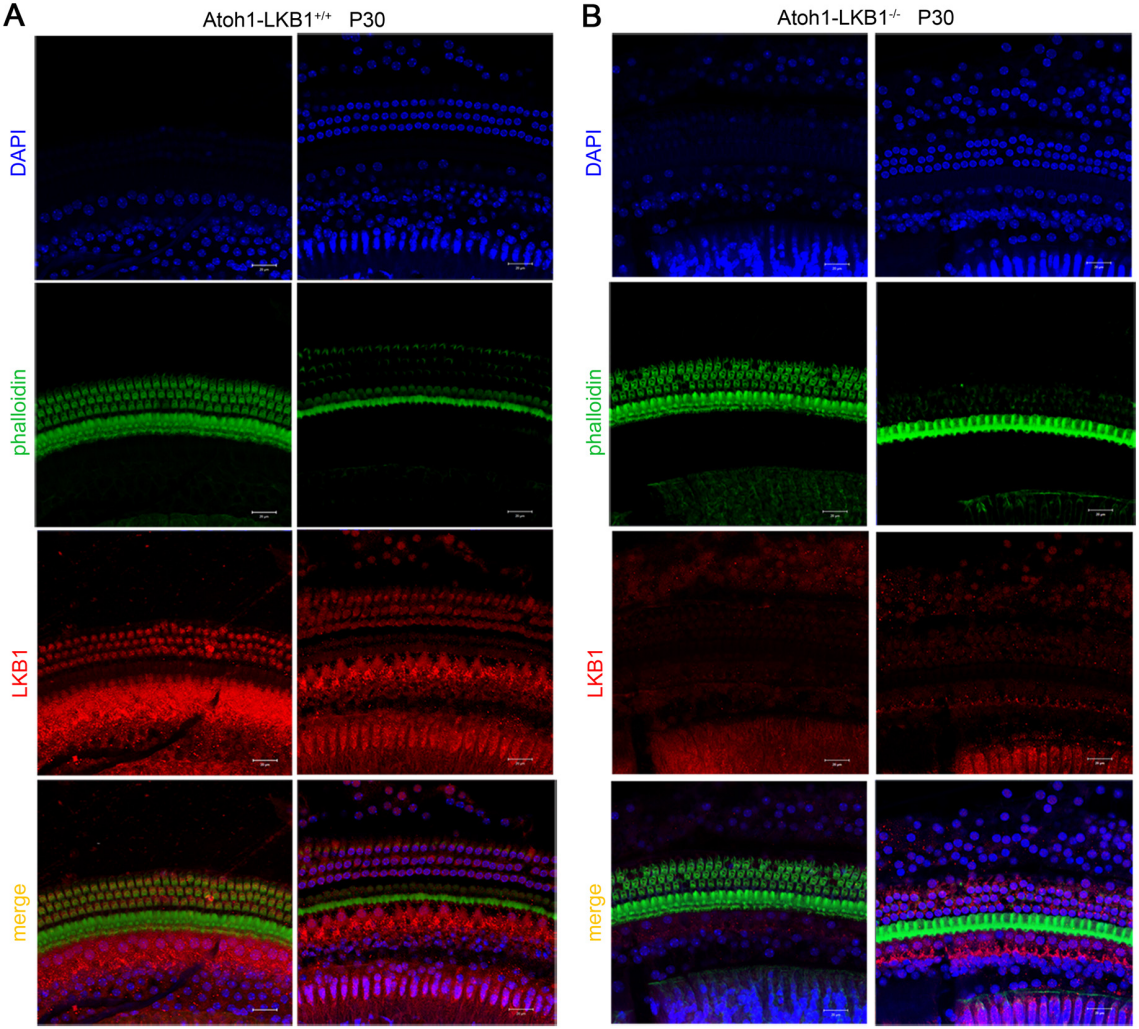
Expression pattern of LKB1 in cochlea hair cells of mice at p30. (A) Confocal images of cochlea hair cells in wild-type mice. LKB1 was expressed from the cuticular plate to the nuclei of hair cells. Left panel: LKB1 expression was detected in the cuticular plate. Right panel: LKB1 expression was detected in the hair cell nuclei. (B) Confocal images of cochlea hair cells in the Atoh1-LKB1^-/-^ mice. LKB1 was deleted in the cochlea hair cells of the inner ear. Left panel: LKB1expression was not detected in the cuticular plate. Right panel: LKB1 expression was not detected in the hair cell nuclei. Green, phalloidin, an F-actin specific dye. Red, LKB1. Bule, Dapi. Scale bar: 20 μm.

To generate LKB1 conditional knockout (Atoh1-LKB1^-/-^) mice, we crossed LKB1^LoxP/LoxP^ mice with Atoh1-Cre mice to inactivate LKB1 specifically in the hair cells of the inner ear and obtained Atoh1-LKB1^-/-^ mice. Atoh1-LKB1^+/+^ mice were used as controls. The genotypes of the pups were identified by PCR analysis (Fig 1A). Whole mount immunostaining was used to examine the expression pattern of LKB1 in the cochlea hair cells, and the results showed a high level of expression in both the nuclei and cytoplasm in the WT mice (Fig 2A). No LKB1 expression was detected in the cochlea hair cells in Atoh1-LKB1^-/-^ mice (Fig 2B). Western blot was used to measure the amount of LKB1 protein in the whole cochlea. A significant decrease of LKB1 protein was observed in Atoh1-LKB1^-/-^ mice compared with that of the WT (Fig 1B). Atoh1-LKB1^-/-^ mice are viable and fertile with no apparent abnormalities in their gross morphology (Fig 1C) except for their decreased weight when compared with their Atoh1-LKB1^+/+^ counterpart (Fig 1D).

### The hearing loss of Atoh1-LKB1^-/-^ mice

**Fig 3:**
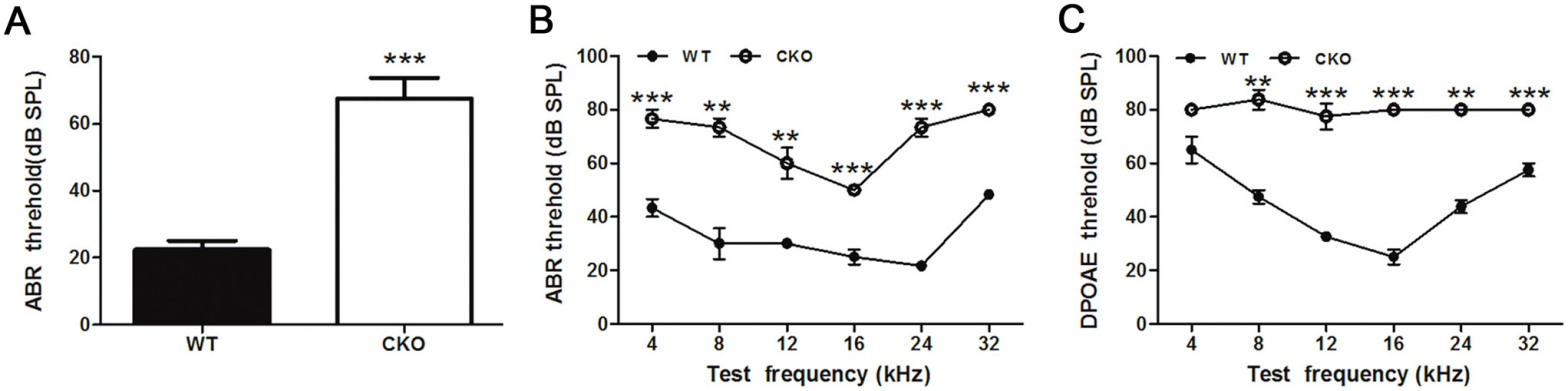
ABR and DPOAE analysis in Atoh1-LKB1^-/-^ and wild-type mice at P30. (A, B) ABR measurements for broadband click (A) and frequency-specific pure tone stimulation (B) of Atoh1-LKB1^-/-^ mice and wild-type mice at P30. (C) DPOAE measurements of Atoh1-LKB1^-/-^ mice and wild-type mice at P30. ABR and DPOAE thresholds in Atoh1-LKB1^-/-^ mice were significantly higher than those in controls. *p<0.05; **p<0.01; ***p<0.001 compared with WT threshold at the corresponding frequency as determined by Student’s t-test; n>4 animals for each experiment and genotype.

To assess the hearing sensitivities of Atoh1-LKB1^-/-^ mice *in vivo*, we tested the ABR thresholds, which consisted of a measurement of the entire hearing cascade. We measured ABR thresholds beginning at P30 to avoid age-related hearing loss. At broadband click, adult Atoh1-LKB1^-/-^ mice exhibited profound hearing loss with a sound pressure level (SPL) of >80 decibels (dB), whereas the control mice showed normal hearing thresholds of 20–30 decibels (dB SPL) (Fig 3A). By varying frequency, cochlear function can be assessed throughout the hearing range. In WT mice, ABR thresholds vary with frequency (Fig 3B) from a sound pressure level (SPL) of 50 dB at 4 kHz (a relatively low frequency for a mouse) to 40 dB at 32 kHz (a relatively high frequency). In LKB1-deficient mice, ABR thresholds were 35 to 60 dB higher than those in wild-type mice (Fig 3B). Deletion of LKB1 resulted in significant threshold elevation in the hearing response. These results showed that the hearing sensitivities of LKB1-deficient mice compared with the control mice were significantly decreased.

To examine OHC function *in vivo*, we measured the distortion product otoacoustic emissions (DPOAEs) in P30 mice. In wild-type mice, DPOAEs thresholds varied with frequency (Fig 3C), from a sound pressure level (SPL) of 60 dB at 4 kHz (a relatively low frequency for a mouse) to 60 dB at 32 kHz (a relatively high frequency). In Atoh1-LKB1^-/-^ mice, DPOAE thresholds were measurable and 15 to 55 dB higher than those in controls at P30 (Fig 3C). These results indicated that *in vivo* OHC function in the Atoh1-LKB1^-/-^ mice was significantly defective in responding to sounds at various intensities compared with the controls.

### Developmental malformation of stereociliary bundles and progressive loss of OHCs in the cochlea of mutant mice

**Fig 4:**
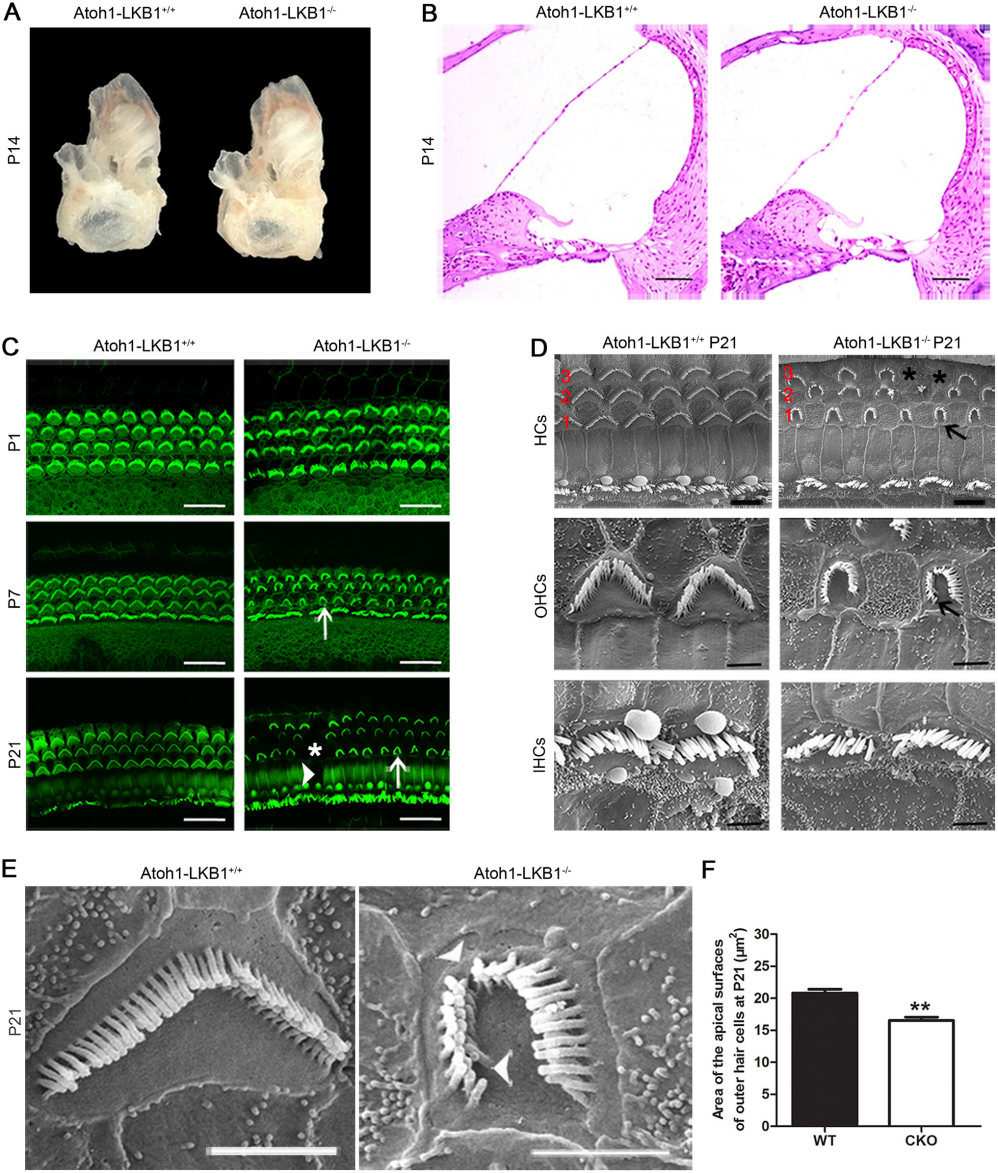
The stereocilia abnormalities of cochlea OHCs in Atoh1-LKB1^-/-^ mice. (A) There was no significant difference in temporal bones between mutant and wild-type mice at P14. (B) Hematoxylin and eosin (HE) staining showed no obvious abnormal morphology of the organ of Corti in Atoh1-LKB1^-/-^ mice. Scale bar: 100 μm. (C) Confocal images of cochlear whole mounts stained with phalloidin. At P1, the hair cell stereocilia showed minor abnormalities in mutant OHCs. The disorder of stereociliary bundles of outer hair cells began to develop and the stereociliary bundle displayed a “U” shape instead of “V” shape in Atoh1-LKB1^-/-^ mice at P7(white arrow), and the phenotypes became worse at P21 (white arrow). Consistent with the sporadic loss of pillar cell (arrowhead), prominent loss of outer hair cells (asterisk) was also observed in Atoh1-LKB1^-/-^ mice at P21. Green, phalloidin, an F-actin specific dye. Scale bars: 20 μm. (D) Scanning electron micrograph (SEM) of hair cell stereocilia in the middle-basal turn of the Atoh1-LKB1^-/-^ and control cochlea at P21. Top panel: Stereociliary bundles of hair cells were abnormal (black arrows), and there was obvious loss of outer hair cells (asterisk) in the Atoh1-LKB1^-/-^ mice. Scale bars: 50 μm. Middle panel: amplified stereocilia of outer hair cells. Stereocilia of outer hair cells were degenerated and an abnormal “U” shape of the stereociliary bundle was displayed in Atoh1-LKB1^-/-^ mice (black arrows). Scale bars: 20 μm. Bottom panel: amplified stereocilia of inner hair cells. A minor disorder in stereociliary bundles was observed in Atoh1-LKB1^-/-^ mice. Scale bars: 20 μm. OHCs: outer hair cells; IHCs: inner hair cells; HCs: hair cells. (E) Scanning electron micrograph (SEM) of stereocilia of the middle-basal turn of outer hair cells in the high resolution. Stereociliary bundles showed degeneration (arrowhead) and an abnormal “U” shape in Atoh1-LKB1^-/-^ mice at P21. Scale bars: 20 μm. (F) Quantitative assessment of the apical surfaces area of outer hair cells in the middle turns of the cochlea at P21. The area of apical surfaces of the hair cells was significantly decreased in the mutant mice compared with that of the controls. Error bars indicate SEM. *p<0.05; **p<0.01; ***p<0.001 compared with the WT group by Student’s t-test; n = 6 hair cells per genotype.

We dissected the cochlea of Atoh1-LKB1^-/-^ mice and observed that the temporal bones of the LKB1-deficient mice had no obvious abnormalities at a gross level (Fig 4A). We also examined the morphology of the cochlea in the inner ear by HE staining. The results also showed no apparent abnormal cochlear morphology in Atoh1-LKB1^-/-^ mice at P14 (Fig 4B).

**Fig 5:**
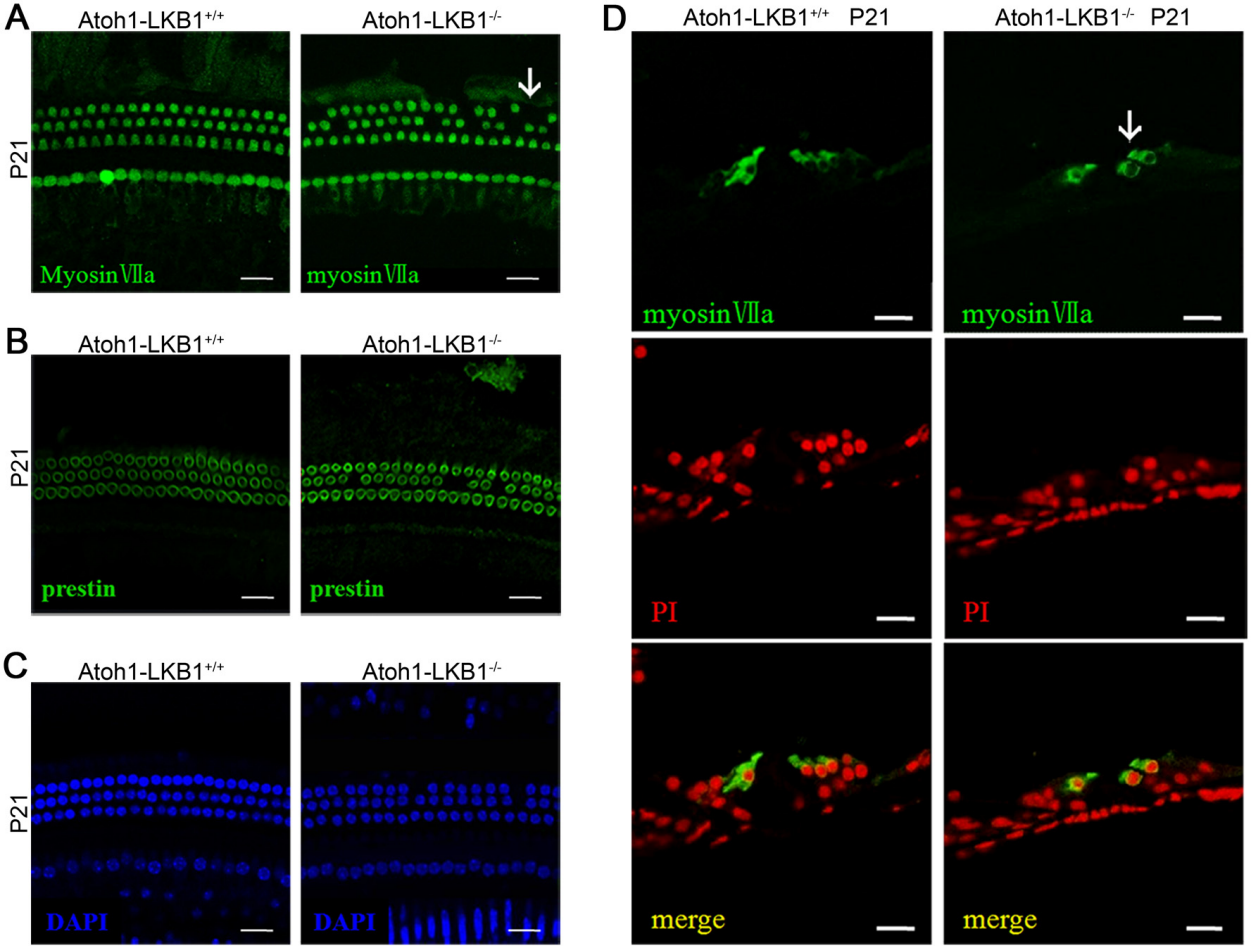
The cell loss of cochlea OHCs in the Atoh1-LKB1^-/-^ mice. Confocal whole-mount images of cochlea in the control and LKB1 mutant cochlea at P21. Hair cells were labeled with the hair cell marker myosin VIIa (green) (A), outer hair cell lateral membrane marker Prestin (green) (B) and the cell nucleus-specific dye DAPI (blue) (C). The morphology of the hair cell cytoplasm, membrane, and nucleus looked normal in the mutant hair cells and controls except for the sporadic loss of outer hair cells. (D) Immunostaining of the cryosections of the cochlea from the midbasal region of control and LKB1 mutant mice at P21 by myosin VIIa (green) and PI (red). The images showed that hair cells in the mutant mice exhibit normal morphology except for the hair cell loss (white arrows). Scale bars: 20 μm.

Using cochlear whole mounts labeled with phalloidin, we subsequently examined the morphology of stereociliary bundles of hair cells in Atoh1-LKB1^-/-^ mice at p1, p7 and p21 (Fig 4C). At P1, the stereociliary bundles of hair cells showed minor abnormalities in morphology (Fig 4C). At p7, the normal “V” shape row of the stereociliary bundle in OHCs now consists of an irregular “U” shape array. However, not hair cell loss was observed in the LKB1 mutant mice (Fig 4C). With further maturation of hair cells, cochlear whole mounts stained with phalloidin displayed severe abnormalities of stereocilia, and the loss of OHCs became obvious in Atoh1-LKB1^-/-^ mice at P21 (Fig 4C). Loss of pillar cells was also observed in the LKB1 mutant mice at P21 (Fig 4C). Scanning electron micrograph showed that stereociliary bundles of OHCs displayed abnormal “U” shapes, and numerous stereocilia were degenerated in the mutant mice at p21 (Fig 4D). A minor disorder in stereociliary bundles was observed and no hair cell loss was found in cochlea inner hair cells (Fig 4D). Higher resolution images of hair cell bundles in mutant mice at P21 confirmed the degeneration of the stereocilia (Fig 4E). In addition to the distorted conformation of the stereocilia, the apical surface area of the outer hair cells in Atoh1-LKB1^-/-^ mice significantly decreased compared with that of the controls (Fig 4F). To examine hair cell loss in detail, we labeled cochlear whole-mount and cryosections with hair cell specific markers, including MyosinVIIa (Fig 5A and 5D) and prestin (Fig 5B). The results showed that Atoh1-LKB1^-/-^ mice suffered from evident loss of OHCs at p21 (Fig 5). The HC loss was quantified at the stages of P21, P30, P60 and P90, and the results showed that the OHCs were lost progressively in the mutant mice (Table 1). We also quantified the presence of HCs in each row. These results showed that the loss of OHCs were most severe in outside row and the least present in the inner side of the OHCs in the Atoh1-LKB1^-/-^ mice (Table 2).

**Table 1:** Percentage of remaining hair cells quantified from three areas of 200μm length near the base of cochlea at four time points.

Genotype	% remaining HC 21 days (base)	% remaining HC 1 month (base)	% remaining HC 2 month (base)	% remaining HC month (base)
	IHC OHC	IHC OHC	IHC OHC	IHC OHC
**WT**	100% 100%	100% 100%	100% 99%	99% 99%
**CKO**	100% 89%	100% 81%	100% 69%	99% 52%

All data are mean form 6 ears from the three wild-type mice (WT) or three Atoh1-LKB1^-/-^ mice (CKO), respectively.

**Table 2:** Percentage of remaining hair cells quantified from three areas of 200μm length near the base in each row of HCs at P30.

Genotype	% remaining OHC Row1 (base)	% remaining OHC Row2 (base)	% remaining OHC Row3 (base)	% remaining IHC (base)
**WT**	100%	100%	100%	100%
**CKO**	98%	82%	63%	100%

All data are mean form 6 ears from the three wild-type mice (WT) or three Atoh1-LKB1^-/-^ mice (CKO), respectively.

### The decrease of phosphorylated ERM proteins in Atoh1-LKB1^-/-^ mice

**Fig 6:**
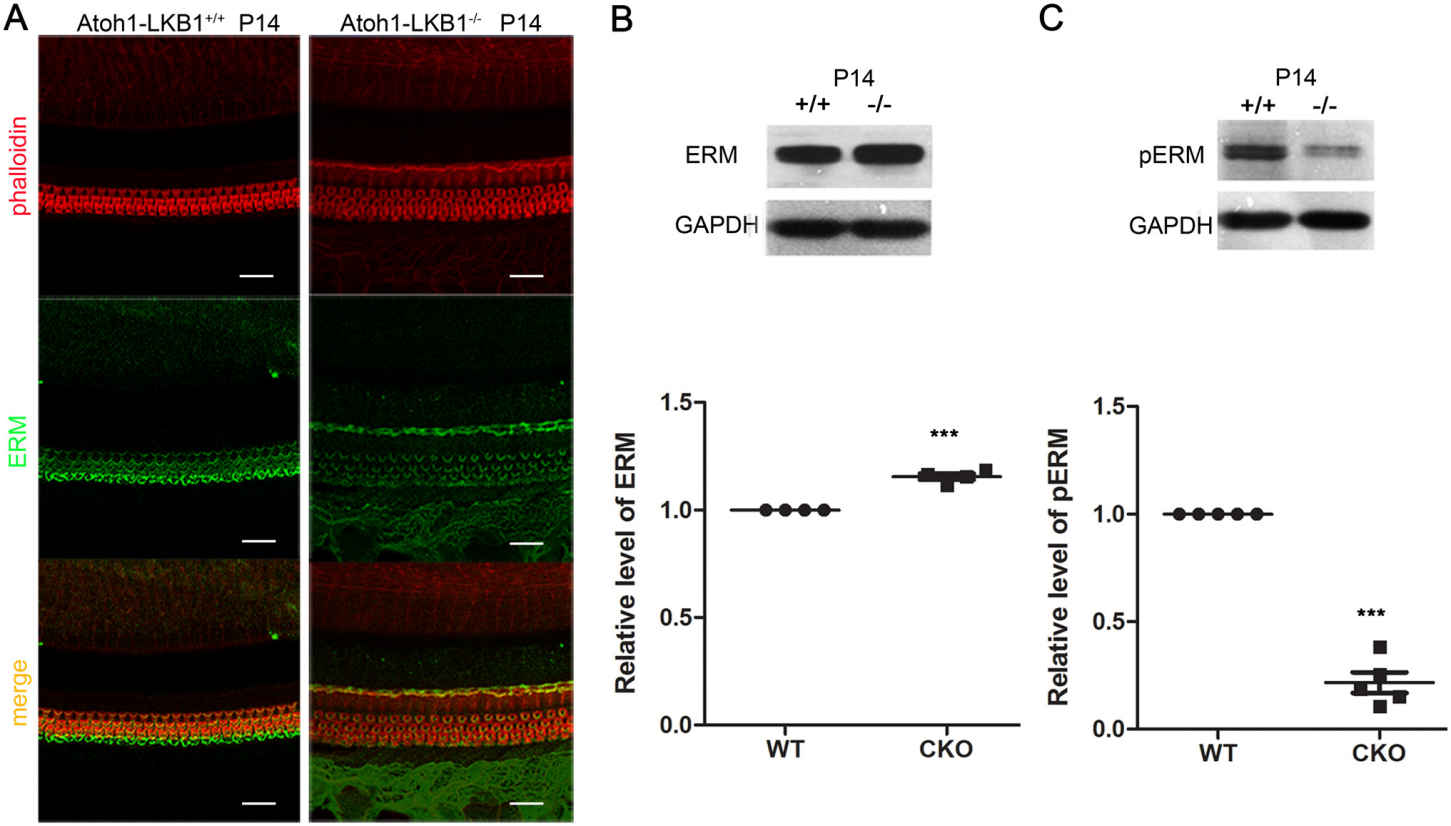
Localization of ERM and the decrease of pERM level in mutant cochlea. (A) Localization of ERM proteins in the hair cells. ERM staining was detected in the bundles of hair cells in both two types of mice at P14. Red, phalloidin; Green, ERM. Scale bars: 20 μm. (B, C) Western blot analysis of the cochlea of mice at P14. Total ERM proteins had a little increase (B), while the level of phosphorylated ERM (pERM) was significantly lower (C) in the Atoh1-LKB1^–/–^ mice (-/-) compared with the controls (+/+). Error bars indicate SEM. *p<0.05; **p<0.01; ***p<0.001 compared with WT by Student’s t-test; n≥4 animals per genotype. Scale bars: 20 μm.

Malformation of the stereocilia of OHCs has been observed in LKB1-deficient mice. It was reported that the stereocilia’s cytoskeletal core is composed of tightly packed and uniformly polarized actin filaments and related actin-binding proteins, which play an important roles in regulating hair cell stereocilia morphogenesis [3, 28, 29]. Therefore, we examined the expression of the ezrin-radixin-moesin (ERM) family proteins, which are the actin-binding proteins that are closely related to the growth and maintenance of stereocilia. The results indicated that ERM proteins were primarily localized to the stereocilia of hair cells in the WT mice (Fig 6A). In the Atoh1-LKB1^-/-^ mice, the location of ERM proteins was similar to that of the controls’ (Fig 6A). The localization of ERM observed in our study was in accordance with the previous reports [30]. By Western blot analysis, Atoh1-LKB1^-/-^ mice showed minimal increase in the total amount of ERM family proteins, but the amount of pERM family proteins was significantly decreased in Atoh1-LKB1^-/-^ mice compared with the levels of WT mice (Fig 6B and 6C). These results indicate that the malformation of stereocilia may be caused by the decrease of actin-binding pERM proteins.

### Mst4 protein was decreased in the inner ear hair cells of the Atoh1-LKB1^-/-^ mice

**Fig 7:**
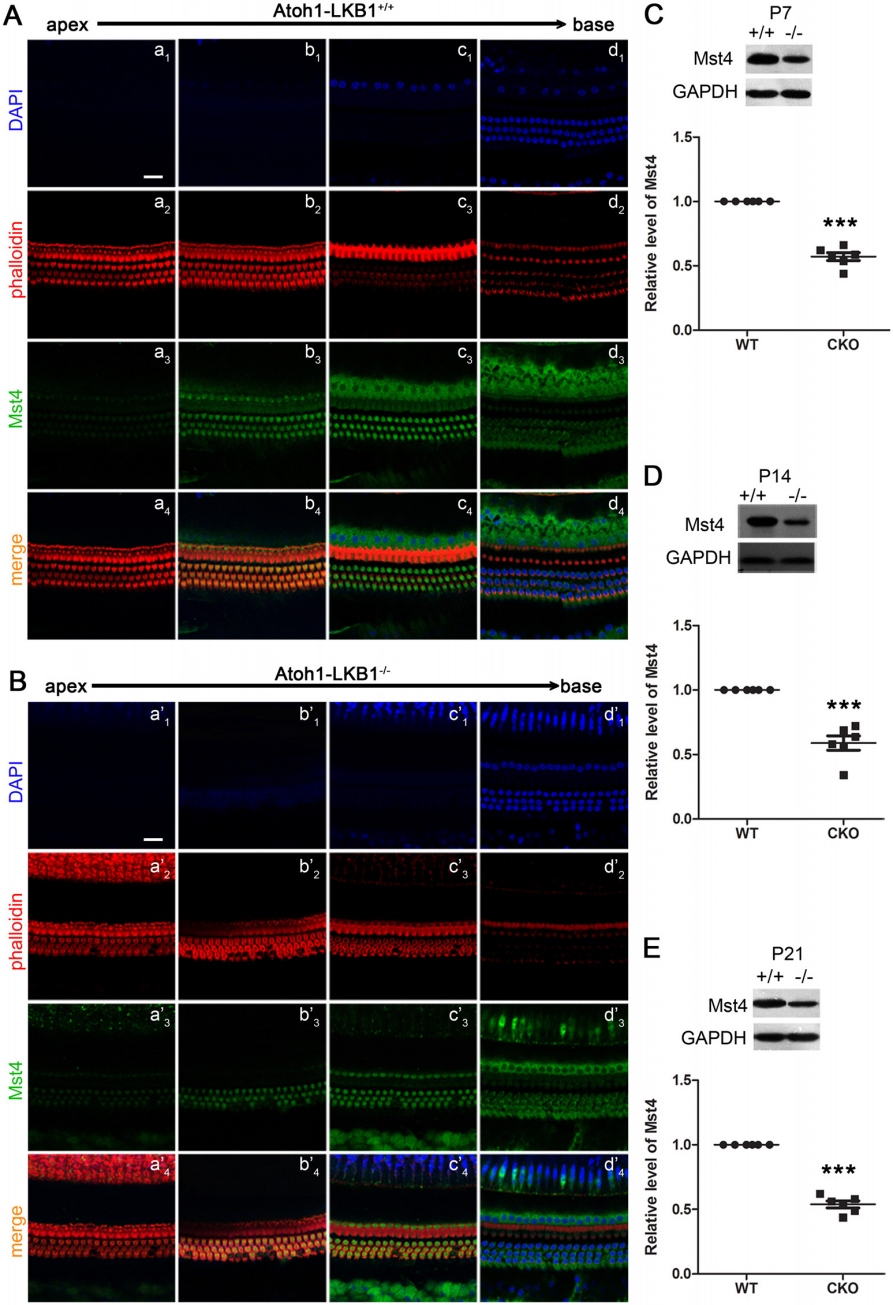
Localization of Mst4 and the decrease of Mst4 in the cochlea of Atoh1-LKB1^-/-^ mice. (A) Immunofluorescence staining of Mst4 was performed using the anti-Mst4 antibody to examine the location of Mst4 in the inner ear hair cells. In the wild-type mice, Mst4 was localized in the cytoplasm (a_3_-d_3_) and cuticular plate (a_2_-d_2_) of hair cells, but not in the stereocilia (a_1_-d_1_) and nucleus (a_4_-d_4_) of hair cells. (B) The localization of Mst4 (a’_1-4_-d’_1–4_) in the hair cells of Atoh1-LKB1^-/-^ mice (a’_1-4_-d’_1–4_) were similar to controls. (C-E) Western blot analysis of the cochlea of mice at P7, P14 and P21 using anti-Mst4 antibody. The Mst4 protein was significantly decreased in the cochleae of Atoh1-LKB1^–/–^mice (-/-) compared with the controls (+/+). Blue, Dapi; Red, phalloidin; Green, Mst4. Scale bars: A, B, 20 μm. Error bars indicate SEM. *p<0.05; **p<0.01; ***p<0.001 compared with WT by Student’s t-test; n = 4 animals per genotype. Scale bars: 20 μm.

Previous reports in the intestinal epithelial cell studies have demonstrated that Mst4, regulated by LKB1, can phosphorylate and activate ezrin, a member of the ERM family of proteins. Activated ERM controls the formation of actin-based cellular brush border whose structure is similar to the stereocilia [31]. Thus, we examined whether Mst4 protein was affected in the inner ear hair cells in Atoh1-LKB1^-/-^ mice. We first examined the expression of the Mst4 protein in cochlea hair cells. Immunostaining analysis showed that Mst4 was localized and enriched in cytoplasm and the cuticular plate of hair cells in WT mice (Fig 7A). A similar Mst4 expression pattern was observed in the LKB1-deficient mice (Fig 7B). Moreover, we examined the level of Mst4 protein by Western blot. The results showed that the amount of Mst4 protein in Atoh1-LKB1^-/-^ mice was significantly lower than that of control at P7, P14 and P21 (Fig 7C, 7D, and 7E). The results demonstrated a significant down-regulation of Mst4 in the LKB1 knockout mice.

## Discussion

Previous studies have reported that LKB1 regulates cell differentiation, migration, proliferation, and regulation of cell polarity establishment during the development of mammals. As an important serine/threonine kinase, LKB1 plays a key role in multiple development signaling pathways and the regulation of the life’s activities. We have detected that LKB1 is highly expressed in the cochlea of the inner ear in mice, and no studies have reported the function of LKB1 during the hearing process. Because the targeted disruption of LKB1 alleles led to embryonic lethality midgestation, we could not investigate the hearing in LKB1 knockout mice. Thus, we have created LKB1 conditional knockout mice (Atoh1-LKB1^-/-^ mice) to study the inner ear.

In our work, we investigated the function of the LKB1 gene in hearing through the analysis of the Atoh1-LKB1^-/-^ mice. The results showed that there were no significant difference between the mutant mice and the controls in gross morphology, except for the decreased weight in the mutants. However, the ABR and DPOAEs thresholds in LKB1 knockout mice were significantly higher than those in the control mice. These findings indicate that the mutant mice experienced hearing loss compared with the controls. The analysis of the hair cell stereociliary bundles in mice’s inner ears at P1 showed minor abnormalities in both OHCs and IHCs. However, at about P7, the outer hair cell stereociliary bundles displayed a conformational “U” shaped abnormality and irregular stair-like structure. After P7, the morphogenesis of stereocilia was more severely damaged in the outer hair cell stereociliary bundles of the mutant group. In our results, we also observed that the sporadic loss of hair cells in LKB1-deficient mice starting at P14. Due to the morphological appearance in chronological order of the disordered stereociliary bundles before hair cell loss, we speculate that there is a direct causal link between the LKB1 gene deficiency and stereociliary bundle abnormalities. In mice, the development and maturation of hair bundles is a complex and precisely regulated process. Cochlear hair cell bundles were reported to continue to develop after birth, and cochlear hair cells become functionally mature at onset of hearing around postnatal 14 days [2, 3]. In the Atoh1-LKB1^-/-^ mice, the severe malformations of hair cell stereocillia occurred before the maturation of the hair cell bundles, and the hair cells suffered from more severe abnormalities after P14. As a result, our findings indicate that LKB1 is required for the development and maintenance of the hair cell stereociliary bundles.

Stereociliary bundles are staircase cytoskeletal microvilli-structures with actin filaments at the core and several actin-binding proteins, such as ERM (ezrin/radixin/moesin) [30, 32, 33], villin [34], fimbrin [35], XIRP2 [8], and FASCIN2 [7]. These actin-binding proteins participate in the assembly of highly organized actin-based structures and maintain the stability of the stereocilia during the development of hair cells [36]. Stereocilia malformation is most likely induced by the abnormality or mutation of actin-binding proteins [37–40]. Among these actin-binding proteins, we first focused on the significant ERM protein family, which is related to bundling actin filaments in the development of hair cell stereocilia [41, 42]. The ERM (ezrin/radixin/moesin) are cross-linkers that connect the plasma membrane and the actin cytoskeleton [43]. ERM proteins are involved in a wide variety of actin-mediated cellular events, including the formation of the stereocilia [30]. In the hearing process, ERM (Ezrin/Radixin/Moesin) proteins are required for stereociliary bundle assembly and maintenance [30, 44], and deficiencies in ERM, especially ezrin and radixin, produce length and shape defects in stereocilia of the inner ear [30].

We first examined ERM protein expression and determined that ERM is primarily enriched in the stereocillia of hair cells in mice. Next, the expression level of ERM and pERM were tested in mutant and wild-type mice. We found a small increase in the total expression level of ERM in the mutant mice. We speculated that the increase of total ERM may be caused by some feedback mechanisms, which need to be identified. Despite a higher presence of ERM in the mutant mice, our results still showed that pERM, which is critically responsible for stereocilia disorganization, was significantly decreased. Therefore, in our mutant mice, the significant decrease of pERM levels potentially resulted in the failure to regulate the actin assembly. The stereocilia cytoskeletal core is composed of tightly packed and uniformly polarized actin filaments, which play an important role in the morphogenesis of hair cell stereocilia. The failure of actin assembly may be a key reason for the deformation of stereocilia in the mutant mice. Thus, these results indicate that the decreased levels of actin-binding pERM proteins may be a primary factor in the stereocilia abnormalities for auditory development and maintenance in the Atoh1-LKB1^-/-^ mice. Taken together, our data indicates that LKB1 may regulate the development and maintenance of stereocilia in the inner ear via the activation of ERM.

Previous studies have demonstrated that LKB1 can act as the upstream regulator protein to indirectly phosphorylate the actin-binding protein Ezrin, a member of ERM proteins through Mst4, and the pERM induced brush border formation in the intestinal epithelial cell [14, 31, 45–47]. The structure of stereocilia is similar to that of the brush border, and both of their primary components are the actin-based cytoskeleton. Thus, we investigated whether the Mst4 was affected by the LKB1 deletion in the Atoh1-LKB1^-/-^ mice. We first examined the Mst4 protein expression in cochlea hair cells. By immunostaining analysis, we found that Mst4 is highly expressed in hair cells. By Western blot analysis, we found that the deletion of LKB1 caused a decreased amount of Mst4 in the Atoh1-LKB1^-/-^ cochlea. It is likely that Mst4 acts an intermediate protein between LKB1 and ERM to phosphorylate ERM during the development and maintenance of stereocilia in hair cells.

In the hearing, hair cell loss is multifactorial in its etiology, affected by both external conditions (ototoxic drugs, noise, etc) and genetic mutations [26, 48–50]. As seen in previous studies, targeted disruption of genes, such as *Cdc42*, *TRIOBP* and other genes, often caused the HC loss by affecting the development and maintenance of actin-based stereocilia in mice [6, 9, 30]. Thus, in our Atoh1-LKB1^-/-^ mice, we proposed that the deletion of LKB1 would result in the disruption of stereociliary bundles, eventually causing the death of hair cells.

**Fig 8:**
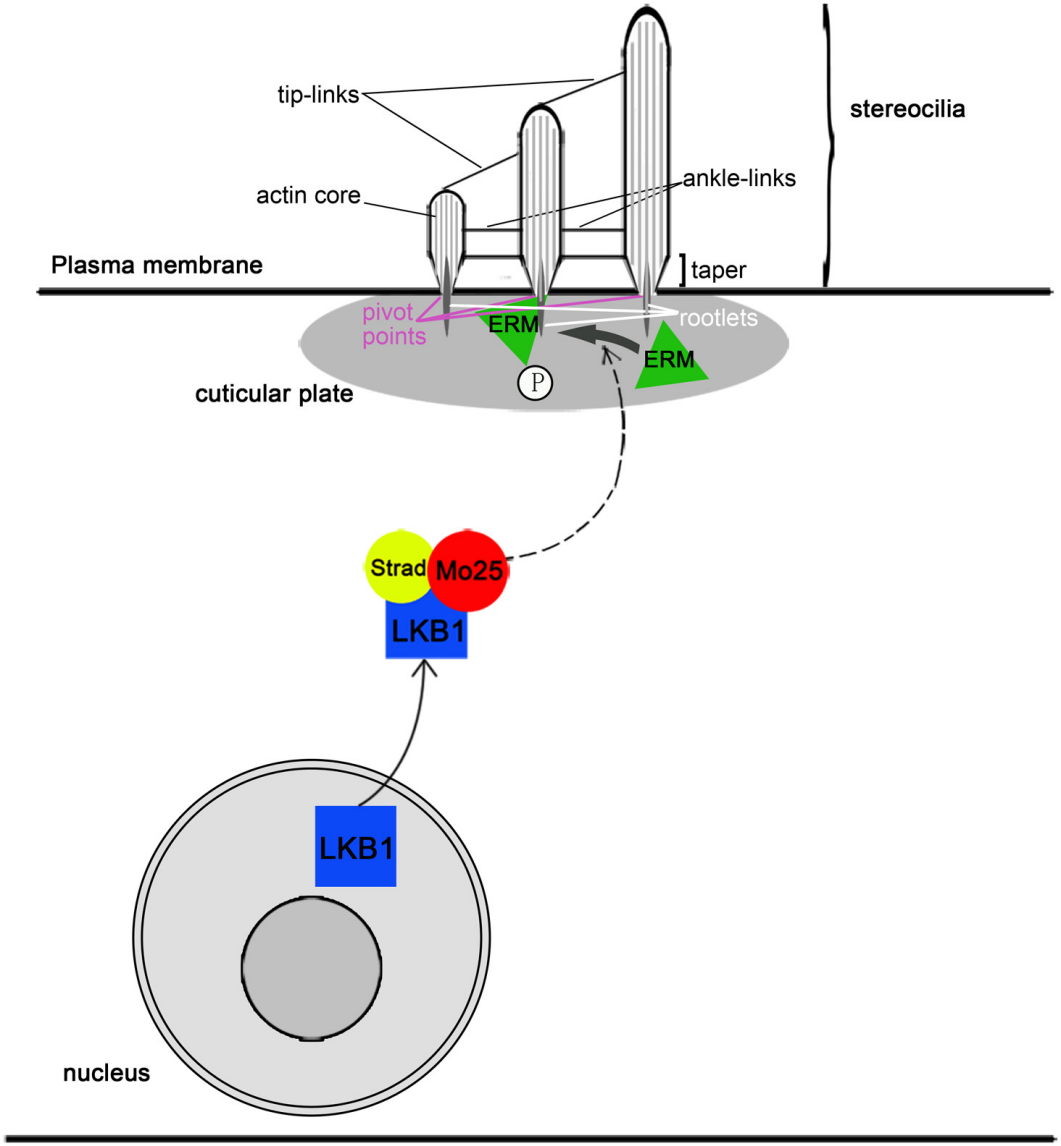
Model of the function of LKB1 in hair cells of the inner ear. LKB1 regulates the development and maintenance of hair cell stereociliary bundles in the inner ear by the activation of ERM. Strad and Mo25 interact and activate LKB1, which may regulate the level of phosphorylated and activated ERM proteins. The phosphorylated ERM protein (p-ERM) controls the actin assembly in the development and maintenance of stereocilia. Deletion of LKB1 in our mutant mice caused a significantly decreased level of pERM, which resulted in abnormal actin assembly. The abnormal development of stereocilia caused hair cell loss and hearing impairment in LKB1-deficienct mice.

In conclusion, we propose that LKB1 regulates the development and maintenance of stereocilia in the inner ear, and this regulation may be through the activation of ERM, which can maintain actin structure and function in the hair cell stereocilia (Fig 8). In our mutant mice model, LKB1 deficiency caused a decrease in the phosphorylation of ERM proteins. Lower pERM levels may be a critical reason for the failure of the actin assembly. Moreover, disorganized actin filaments resulted in disordered stereocilia, which led to the disruption of the stereocilia function. Damaged stereocilia induces rapid hair cells death, and loss of hair cells was observed. Eventually, all abnormalities in the hair cells resulted in hearing impairment. Taken together, our data reveal a novel understanding of the genetic control of LKB1 in development and maintenance of stereociliary bundle.

LKB1 is involved in a variety of human diseases such as Peutz-Jeghers Syndrome and various types of cancers. The genetic component linking LKB1 and hearing loss show clinical significance of testing patients who are diagnosed with Peutz-Jegher Syndrome for hearing impairment. In addition, in patients who suffer from hear loss, screening for LKB1 mutation could reveal a genetic disposition for the condition. Not only can Atoh1-LKB1^-/-^ mice be used to examine the LKB1 gene function during the development and maturation of the hearing process, but they will also provide an ideal mouse model to study therapeutic intervention for deafness in the hearing field.
